# CNN-LSTM Model for Recognizing Video-Recorded Actions Performed in a Traditional Chinese Exercise

**DOI:** 10.1109/JTEHM.2023.3282245

**Published:** 2023-06-02

**Authors:** Jing Chen, Jiping Wang, Qun Yuan, Zhao Yang

**Affiliations:** School of Electronic and Information EngineeringSuzhou University of Science and Technology 66339 Suzhou 215009 China; Suzhou Institute of Biomedical Engineering and Technology 165085 Suzhou 215000 China; Department of Respiratory MedicineSuzhou Hospital, Affiliated Hospital of Medical School, Nanjing University 12581 Suzhou 215163 China

**Keywords:** Action recognition, CNN, geometric feature extraction, LSTM, video processing, Clinical and Translational Impact Statement-The proposed algorithm can recognize the complicated actions in rehabilitation training and thus has the potential to realize intelligent rehabilitation assessment for home applications

## Abstract

Identifying human actions from video data is an important problem in the fields of intelligent rehabilitation assessment. Motion feature extraction and pattern recognition are the two key procedures to achieve such goals. Traditional action recognition models are usually based on the geometric features manually extracted from video frames, which are however difficult to adapt to complex scenarios and cannot achieve high-precision recognition and robustness. We investigate a motion recognition model and apply it to recognize the sequence of complicated actions of a traditional Chinese exercise (ie, Baduanjin). We first developed a combined convolutional neural network (CNN) and long short-term memory (LSTM) model for recognizing the sequence of actions captured in video frames, and applied it to recognize the actions of Baduanjin. Moreover, this method has been compared with the traditional action recognition model based on geometric motion features in which Openpose is used to identify the joint positions in the skeletons. Its performance of high recognition accuracy has been verified on the testing video dataset, containing the video clips from 18 different practicers. The CNN-LSTM recognition model achieved 96.43% accuracy on the testing set; while those manually extracted features in the traditional action recognition model were only able to achieve 66.07% classification accuracy on the testing video dataset. The abstract image features extracted by the CNN module are more effective on improving the classification accuracy of the LSTM model. The proposed CNN-LSTM based method can be a useful tool in recognizing the complicated actions.

## I. Introduction

Identifying human activities from raw observation data has long been a question of great interest in intelligent rehabilitation, health surveillance, medical caregivers, training recovery and skill assessment [1], [2], [3], [4]. Mainstream research methods to solve such problems can be classified into two categories, that is, recognition methods based on video data and those based on time series data obtained via wearable sensor [5]. Human activity recognition based on video data utilizes image or video processing technology to realize human behavior recognition and specific target detection by analyzing the image set or video clips of human motion obtained via imaging sensors [6], [7], [8]. Human activity recognition based on wearable sensors aims at seeking the deep knowledge contained in human behavior from the sensor data. Such wearable devices acquire human motion data through built-in transducers, e.g., accelerometers. Each channel of such a sensor outputs a one-dimensional time series; and thus, the output signals of all sensors form multidimensional time series [9]. No matter which method is opted, the next key task is to design appropriate model to process the data to achieve effective feature representation and classification, so as to realize human activity recognition [10], [11], [12].

Along this track, efforts have recently been made to recognize the actions in Yoga [3]. Similar to Yoga, Baduanjin is a traditional Chinese exercise with scientifically arranged intensity and finely orchestrated movements [13], [14]. Modern studies have confirmed that Baduanjin can improve the function of neurohumoral regulation and strengthen blood circulation, and has a good regulatory effect on nervous system, cardiovascular system, digestive system, respiratory system and motor organs [15], [16], [17]. In the case that it is practiced correctly, Baduanjin can strengthen the body, regulate the mood and promote the recovery from chronic diseases. As a part of traditional Chinese medicine (TCM) health preservation and therapy, Baduanjin has been promoted to the world by the General Administration of sports of China for its good physical exercise effect [18], [19], [20], [21], [22]. However, despite its benefits and popularity, Baduanjin action recognition has not yet been studied in the field of human activity recognition.

Traditionally, rehabilitation exercise therapy for chronic diseases is usually carried out in a formal rehabilitation center or clinical environment, and requires the direct supervision of professional physiotherapists [23]. The computer-assisted self-training system for rehabilitation training is a feasible alternative. It can guide participants to exercise, improve their performance and reduce injuries, and also reduce the burden of medical resources.

In this literature, some human activity recognition systems based on wearable sensors have been developed [24], [25], [26], [27]. Although the accuracy of collected motion information by wearable devices is guaranteed, there are some problems such as inconvenient wearing and vulnerability to damage. Some people cannot wear it for a long time because of the special structures of these devices, which make them feel uncomfortable. In addition, because wearable devices are usually connected with other portable devices via Bluetooth, it is inevitable to leak the privacy of users in the process of uploading personal data. On the other hand, with the promotion of mobile internet applications and the popularity of smart phones, digital cameras and other imaging devices, video data are much more easily accessible, which can provide a wealth of human action information.

Feature extraction and classification are two main procedures in video action recognition. Klishkovskaia et. al. [28] have developed three posture classification algorithms based on joint data. The algorithms are based on the total errors of both the vector lengths and the angles, and the multiplication of these two errors. The proposed algorithm has been shown to have good effect on the classification of simple human postures. However, the effect on more complex movements such as Baduanjin still remains to be further explored. Hu et. al. [29] have proposed an interactive retrieval system based on the nonlinear time warping algorithm to retrieve actions similar to the query motion performed by users. However, a depth sensor-based camera is needed, which is usually unavailable to users. On the other hand, deep convolutional neural networks (CNN) are widely used in feature extraction and classification [30]. Andrej et. al. [31] proposed a CNN based model, and proposed late fusion, early fusion and slow fusion strategies to fuse time information. However, this can only obtain limited time information. Tran et. al. [32] proposed a model based on a three-dimensional CNN (3DCNN), which integrates time information on the basis of a two-dimensional CNN. However, this model consumes more spatial-temporal resources and converges slowly.

On the other hand, thanks to the excellent performance of CNN in the field of image recognition and the outstanding results of long short-term memory network (LSTM) in modeling time series data, efforts have been paid to combine these two algorithms to build a video-based action recognition model [33], [34]. Donahue et. al. [35] proposed a LRCN (Long-term recurrent Convolutional Networks) model based on CNN and LSTM. The model first extracts spatial information through the CNN, then extracts time information through the LSTM network, and finally outputs the results after a softmax module. Based on the recent success of recurrent neural networks in modeling dynamic time series, Francisco et. al. [36] proposed a generic deep framework for activity recognition based on convolutional and LSTM recurrent units. Their results showed that the framework outperforms deep non-recurrent networks on the challenge dataset by 4% on average. Subsequently, many improvements were made to the structure of the CNN-LSTM model, such as TS-LSTM, TS-LSTM has made progress in time related modeling [37].

In light of this, we propose in this work a motion recognition model based on LSTM and a popular CNN model, the VGG16 (VGG ConvNet configuration D) architecture proposed by the Visual Geometry Group [38], in view of its excellent learning ability of complex features. Specifically, the pre-trained VGG16 network is used to extract the feature vector sequence of the video frames. Therefore, the newly developed model is abbreviated as CNN-LSTM model. On the other hand, the recent research along this track has been carried out through training deep learning human behavior recognition systems for simple daily activities such as walking, standing, lying, waving, and raising legs and so on. Such models and algorithms have not yet been applied to recognize the actions in a more complicated exercise like Baduanjin, which contains eight finely orchestrated movements. This work is attempted to abridge this gap, by developing and testing the CNN-LSTM model to recognize the actions in Baduanjin. Moreover, using a Baduanjin action data set, we will also compare the newly developed CNN-LSTM model with that based on the traditional manual feature extraction method.

In summary, there are two main contributions in this work. First, a video-based human action recognition model is proposed and especially trained to recognize Baduanjin actions, using the VGG16 and LSTM network. Second, the performance of the Baduanjin action recognition model based on the traditional manual feature extraction procedures is also established, and compared with that of the newly developed CNN-LSTM method.

## II. Model Architecture and Recognition Method

### A. Model Architecture

**Fig. 1.**
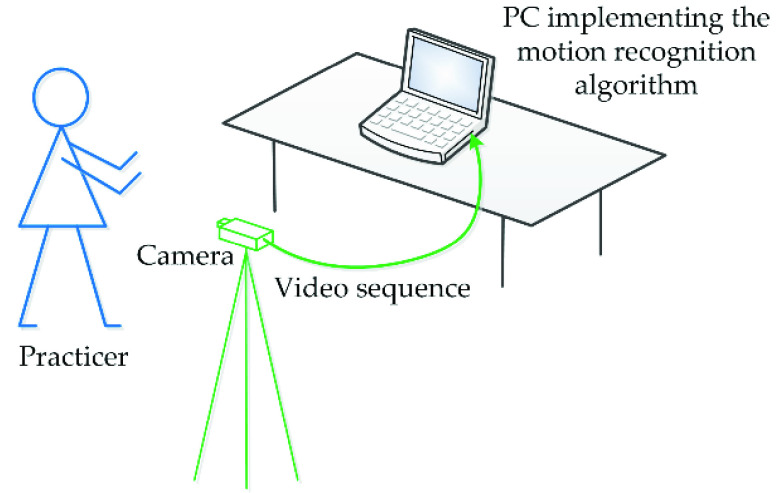
Experimental setup to collect and recognize practicers’ actions.

**Fig. 2.**
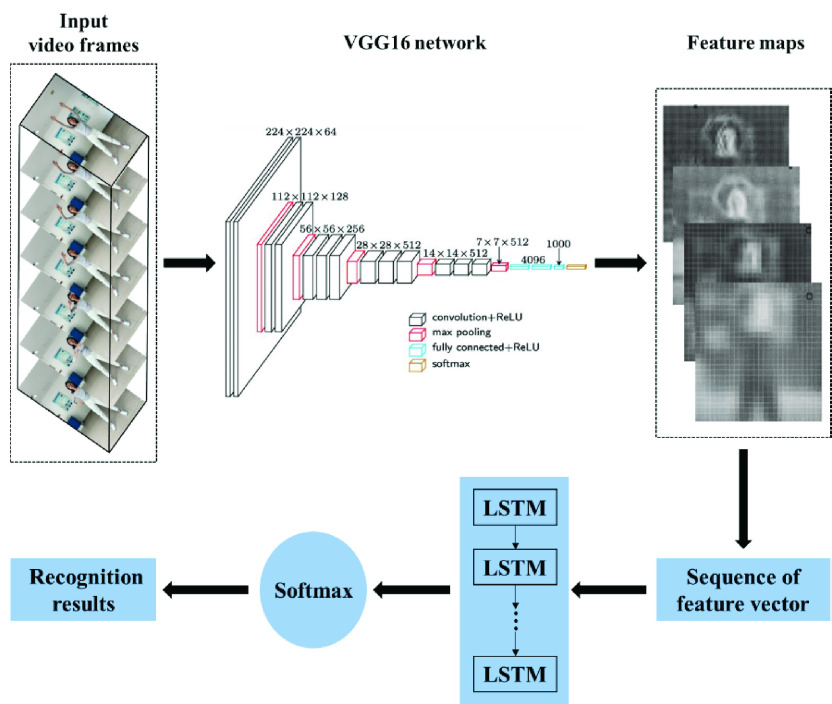
CNN-LSTM model architecture of for Baduanjin action recognition.

The experimental setup to collect and recognize Baduanjin practicers’ actions is shown in Fig. 1, including an imaging device and a PC implementing the proposed model. More specifically, the CNN-LSTM based Baduanjin action recognition method can be divided into the following steps as shown in Fig. 2. First, video data in the form of a sequence of RGB image frames are collected by the setup shown in Fig. 1, which can either be from a real-time process running in parallel with the action recognition, or be offline recorded videos. Second, the feature vector sequence is extracted from the video frames by the pre-trained VGG16 network, which is then fed into the LSTM network to further analyze their changes over time. Finally, the identified Baduanjin action categories are produced.

**Fig. 3.**
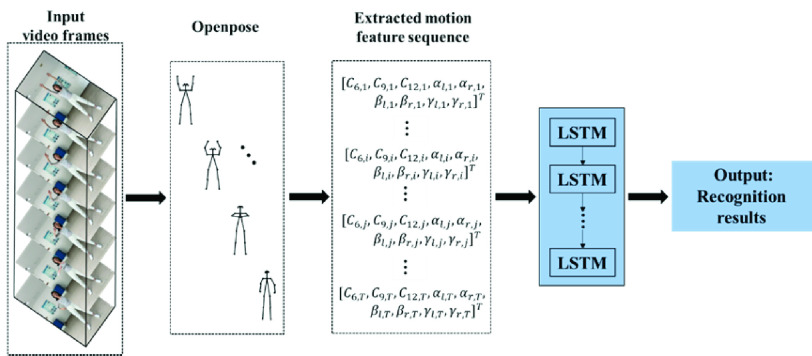
Traditional manual feature extraction-based model architecture for Baduanjin action recognition.

On the other hand, the traditional manual feature extraction based on the geometry of the detected skeletons is also considered for comparison with the CNN-based feature extraction. To this end, Openpose is used to identify the joint positions in the skeletons [39]. For a fair comparison, a classifier based on LSTM is then also applied for human pose recognition. This model architecture is shown in Fig. 3.

### B. Geometric Motion Features Extracted from Skeleton Data Detected By OPENPOSE

Traditional motion recognition technology mainly depends on manual feature extraction. The selection of features generally depends on the domain knowledge of experts [40]. For human action recognition, skeleton point extraction is the first step of our pipeline, which is realized via Openpose, a widely used open-source library for key point detection. It is able to identify the joint position using partial confidence maps and part affinity fields, followed by binary matching and parsing [41].

**Fig. 4.**
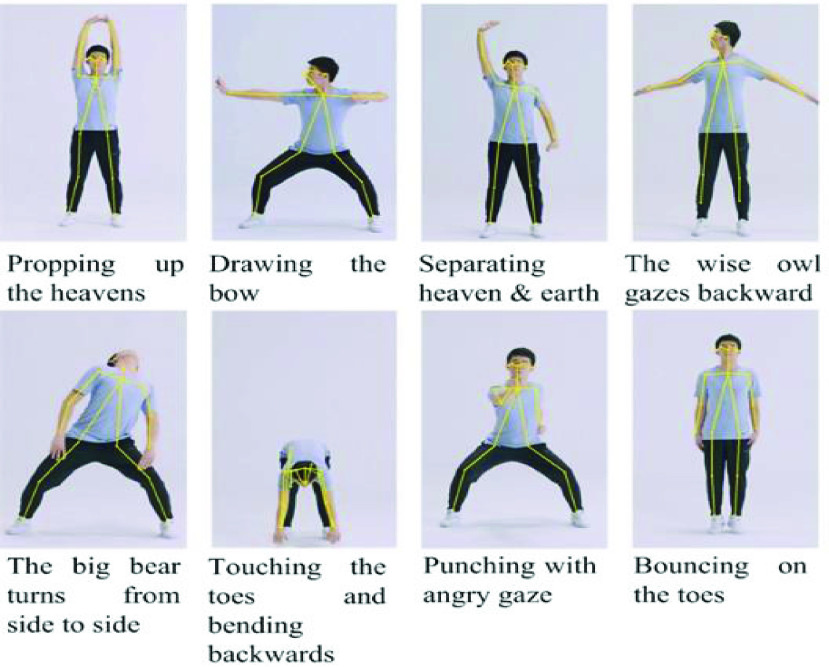
Poses and corresponding extracted skeleton structures during the Baduanjin movements.

**Fig. 5.**
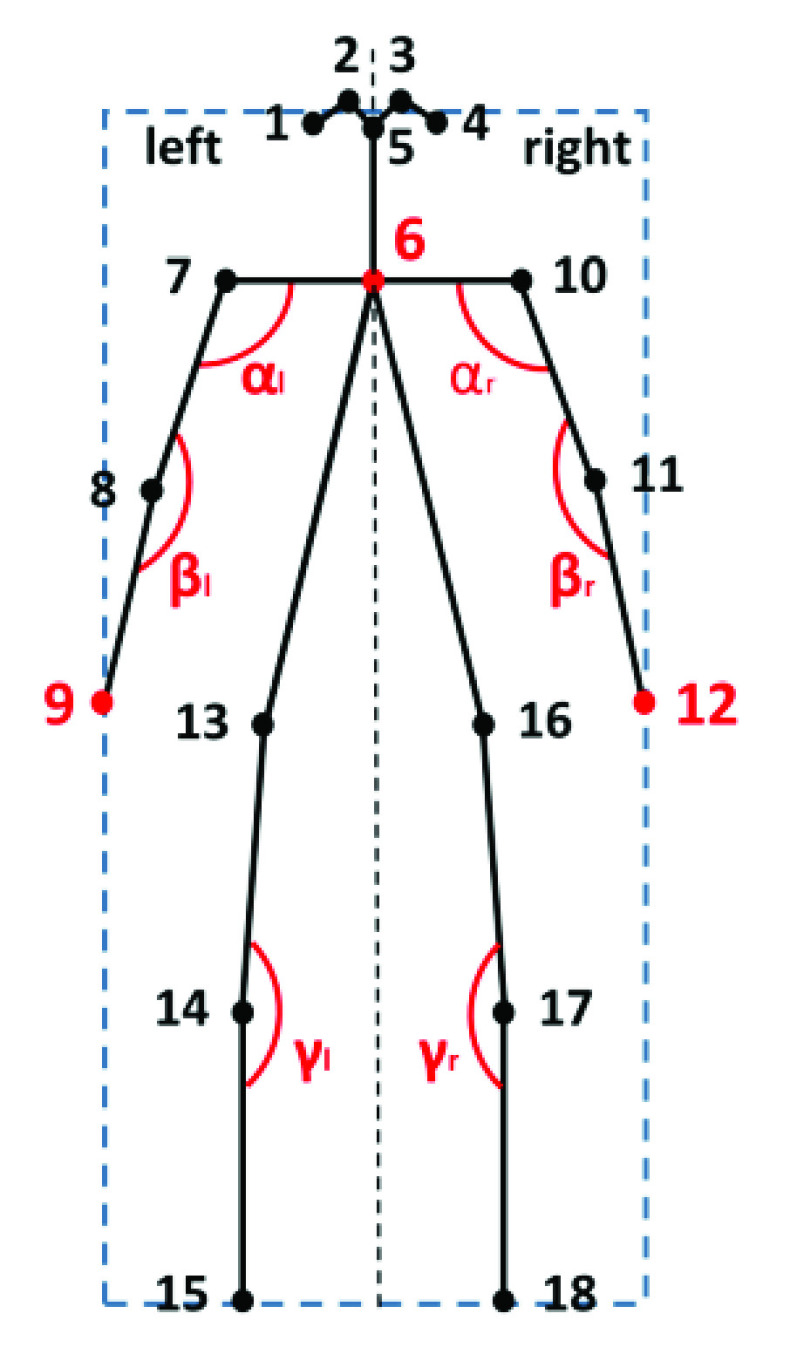
Manual feature extraction, with the stick model showing body segments, joints and the selected motion features.

More specifically, from the 18 key point coordinates tracked by Openpose, the key node positions and joint angles in each video frame are extracted and calculated. The detailed Baduanjin poses are described in Fig. 4. The feature extraction procedure aims to obtain posture-related features from the joint positions and angles provided by the bone tracking system. According to the suggestions of professional physiotherapists, some typical joint angles and position trajectories are selected to distinguish among different Baduanjin movements. Fig. 5 shows the stick model for body segments and highlights the features extracted for Baduanjin movements.

Specifically, the initial skeleton data are preprocessed for the spatial alignment between a skeleton instance and its template counterpart. The coordinate system is established with the center of gravity of the human skeleton as the origin. The coordinates of each joint point in the human skeleton are calibrated. Denote the coordinates of the center of gravity of the human skeleton as $(x^{0}_{c},y^{0}_{c})$. Then, $x^{0}_{c}=(x_{6}+x_{13}+x_{16})/3$ and $y^{0}_{c}=(y_{6}+y_{13}+y_{16})/3$. The coordinates of the joint points before and after the calibration can be converted as follows:

\begin{align*} \begin{cases} \displaystyle x_{i}=x_{i}^{0}-x_{c}^{0}\\ \displaystyle y_{i}=y_{i}^{0}-y_{c}^{0} \end{cases} \tag{1}\end{align*}

where $(x_{i}^{0},y_{i}^{0})$ and $(x_{i},y_{i})$ are the coordinate of the $i$-th joint point before and after calibration.

Furthermore, through the geometric analysis of the poses of the human body with open arms facing the camera, the minimum circumscribed rectangle of the skeleton in a video frame can be calculated as follows:

\begin{align*} T=&\frac {1}{4}\sum \limits _{i=1}^{4} {y_{i}} \\ B=&\frac {1}{2}(y_{15} +y_{18}) \\ L=&x_{9} \\ R=&x_{12} \tag{2}\end{align*}

Then, the width $W$ and height $H$ of the minimum circumscribed rectangle are respectively:

\begin{align*} H=&B-T \\ W=&R-L \tag{3}\end{align*}

Let $r_{x}$ and $r_{y}$ respectively be the ratio of the standard width and height extracted from the poses of an expert of this exercise to those extracted from a practicer’s poses in the $x$ and $y$ direction, that is,

\begin{align*} r_{x}=&\frac {H_{0}}{H}=\frac {H_{0}}{\frac {1}{2}(y_{15}+y_{18})-\frac {1}{4}\sum _{i=1}^{4}y_{i}} \\ r_{y}=&\frac {W_{0}}{W}=\frac {W_{0}}{x_{12}-x_{9}} \tag{4}\end{align*}

Here, $H_{0}$ and $W_{0}$ are the standard height and width extracted from the poses of an expert of this exercise respectively.

In summary, nine features including joint point positions or angles as shown in Fig. 3 are extracted from the preprocessed skeleton data as action classification features. These features are listed in Table 1. It is worth mentioning that the symmetry of the practicer’s skeleton can also be analyzed based on these features. The mismatches with the standard skeleton sequence are marked, which can help the practicer adapt to the best positions and directions.

**TABLE 1:** The Selected Typical Joint Angles and Position Trajectories Based on the Skeleton Geometry Shown in Fig. 5, Where CI, I=6, 9, 12, are the Coordinates of Respectively the 6-th, 9-th and 12-th Point

No.	FEATURE TYPE	Symbol	Description
1	Key point	$C_{6}$	neck
2	Key point	$C_{9}$	Left wrist
3	Key point	$C_{12}$	Right wrist
4	Key angle	$\alpha _{1}$	Left shoulder joint angles
5	Key angle	$\alpha _{F}$	Right shoulder joint angles
6	Key angle	$\beta _{ I }$	Left elbow joint angles
7	Key angle	$\beta _{r}$	Right elbow joint angles
8	Key angle	$\gamma _{0}$	Left knee joint angles
9	Key angle	$\gamma _{ r }$	Right knee joint angles

### C. Video Feature Vector Sequence Extraction Based on CNN

The proposed Baduanjin action recognition algorithm firstly uses the pre-trained VGG16 network to extract the feature vector sequence from every video frame collected by the RGB camera. The feature vector sequence is then sent to the trained LSTM model to realize the recognition of Baduanjin actions.

More specifically, since the CNN does not support video inputs, each video clip is first intercepted and saved in the original frame rate by sparse down sampling. To see this, let a video clip be denoted as $v_{m}=\{f_{1}^{m},f_{2}^{m},\ldots,f_{T}^{m}\}$, where $f_{T}^{m}$ represents the $t$ -th frame in the $m$ -th video clip $v_{m}$; and $T$ is the total number of frames of the video clip. After sampling, the picture set denoted by $v'_{m}=\{f_{1}^{m},f_{2}^{m},\ldots,f_{s}^{m}\}$ with length s is obtained. Average down sampling method is adopted, that is, sampling every $\lfloor \frac {T}{s}\rfloor $ from the first frame. For $s>T$, the original video length $T$ remains unchanged.

A CNN is generally composed of convolution layers, pooling layers, full connection layers and softmax layers. Likewise, VGG16 is built by repeatedly adding 3-by-3 convolution kernels and 2-by-2 pooling kernels to achieve feature extraction. The network can not only simulate the effect of larger receptive field and increase the number of feature map channels, but also achieve better extraction [42]. This study uses the VGG16 network trained by the ImageNet dataset to extract feature vector sequences from the test video frames as shown in Fig. 2. After the input video is decomposed into a sequence of frames by the down sampling method, the features are extracted and optimized through 13 convolution layers and 5 pooling layers in the VGG16 net.

### D. Video Action Recognition Based on LSTM

The movements of Baduanjin are dynamically arranged in a temporal order. To process these time series data, LSTM is a better option than general neural networks, due to its recurrent connections. More specifically, LSTM introduces memory units to expand a cyclic convolution network, controls the state and behavior of each memory unit with the concept of gate, and can thus establish the dependency between related information on a longer time scale. Therefore, the dependency of the sequential Baduanjin movements on each other can be captured by a LSTM network.

**Fig. 6.**
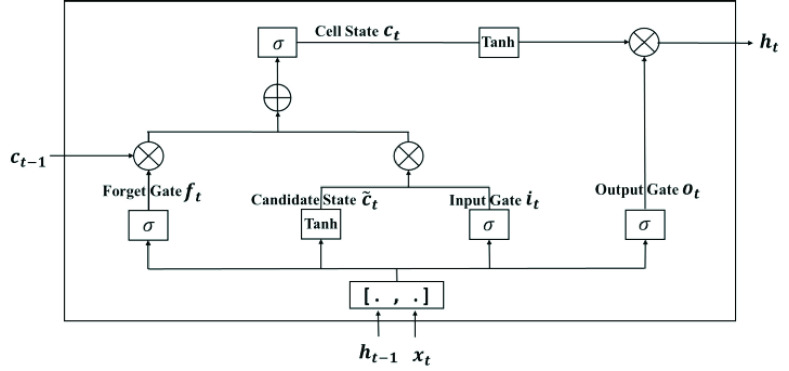
The structure of a LSTM cell.

Fig. 6 shows the structure of a single LSTM cell applied in this work [43]. The inputs of the cell include the input $x_{t}$ at the current time $t$, the output $h_{t-1}$ and the memory state $c_{t-1}$ at the last time instant ${t-1}$. The outputs at the current time $t$ include both $h_{t}$ and $c_{t}$.

The LSTM controls the memory unit through the input gate and forgetting gate, and combines the output gate to describe the long-distance dependence more effectively. The mathematical operations of the input gate, forgetting gate and output gate can be respectively written as follows:

\begin{align*} i_{t}=&\sigma ({\mathrm{\mathbf W}}_{i} \cdot \left [{ {h_{t-1},x_{t}} }\right]+b_{i}) \\ f_{t}=&\sigma ({\mathrm{\mathbf W}}_{f} \cdot \left [{ {h_{t-1},x_{t}} }\right]+b_{f}) \\ o_{t}=&\sigma ({\mathrm{\mathbf W}}_{o} \cdot \left [{ {h_{t-1},x_{t}} }\right]+b_{o}) \tag{5}\end{align*}

where $\boldsymbol {W}_{i}, \boldsymbol {W}_{f}$ and $\boldsymbol {W}_{o}$ are the weight matrices of input gate, forgetting gate and output gate respectively; $b_{i}$, $b_{f}$ and $b_{o}$ are the offsets of input gate, forgetting gate and output gate respectively; represents the sigmoid function, whose output is between 0 and 1. The output of the LSTM cell is obtained by sequentially performing the calculations in the memory unit and output gate as follows:

\begin{align*} \tilde {c}_{t}=&\tanh ({\mathrm{\mathbf W}}_{c} \cdot h_{t-1} +{\mathrm{\mathbf W}}_{c} \cdot x_{t} +b_{c}) \\ c_{t}=&f_{t} \times c_{t-1} +i_{t} \times \tilde {c}_{t} \\ h_{t}=&o_{t} \times \tanh (c_{t}) \tag{6}\end{align*}

where $\tilde {c}_{t}$ is the candidate state at time $t$; $\boldsymbol {W}_{c}$ is the weight matrix of the candidate states; ${b}_{c}$ is the offset of the candidate states; ${c}_{t}$ is the cell state of time ${t}$; and ${h}_{t}$ is the final output at time ${t}$.

## III. Results

### A. Dataset

Since there is no public image dataset of Baduanjin for pattern recognition yet, we collected the videos of 18 people (11 men and 7 women) performing the entire routines of the eight poses individually. The original videos were in the MP4 format, with a resolution of $1640\times480$ pixels. All poses were performed at a distance of 2 $\sim $3 meters in front of the camera. The practicers performed every pose with all possible changes. All poses were collected for more than 10 s at a rate of 30 frames per second (FPS) in an indoor environment. The total number of video clips for each individual practicer was 459, amounting to a total of more than 137700 frames. Table 2 describes the collected dataset, including the number of persons and the number of videos per pose. We used the videos of distinct subjects for training and testing sets with a 3:1 division at the video level.

**TABLE 2:** The Details of the Collected Data Set

No.	Pose name	No. of persons	No. of videos
1	Propping up the heavens	18	57
2	Drawing the bow	18	57
3	Separating heaven & earth	18	58
4	The wise owl gazes backward	18	56
5	The big bear turns from side to side	18	57
6	Touching the toes and bending backwards	18	58
7	Punching with angry gaze	18	57
8	Bouncing on the toes	18	59
	Total number of videos		459

The performed poses in the datasets varied greatly from person to person even for a same pose, due to the following reasons. First, the participants performed the same movements at different speeds in different repetitions. Second, the participants were of different heights and shapes. Third, the participants performed the routines in different scenes. These variations made the video data more complicated, and necessitated the robustness of the recognition method.

### B. Evaluation Criterion

The accuracy of the recognition method was calculated as

\begin{equation*} accuracy=\frac {TR}{TR+FR} \tag{7}\end{equation*}

where ${TR}$ and $FR$ denote the number of samples respectively with successful and failed recognition. Furthermore, to demonstrate the advantage of the proposed Baduanjin action recognition method for different poses, the two methods based on different feature extraction methods as described in Sec II. were compared. On this basis, the confusion matrix is used to measure the performance of the CNN-LSTM based Baduanjin action recognition model. The prediction accuracy of each category is on the diagonal of the normalized confusion matrix. Note that a good classification model shall lead to a normalized confusion matrix, with diagonal elements as close to 1 as possible, and with off-diagonal elements as close to 0 as possible.

### C. Results

**Fig. 7.**
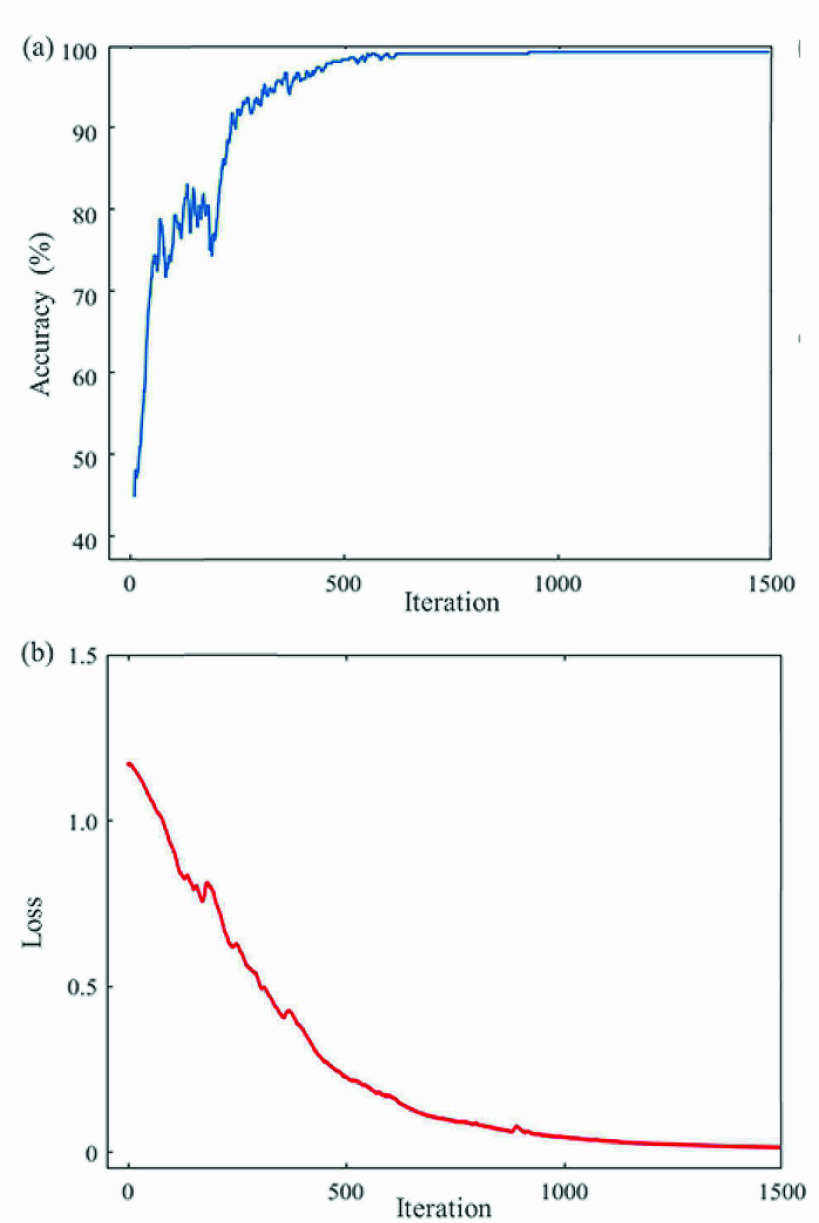
Training process of the CNN-LSTM model: (a) and (b) are respectively the accuracy curve and loss curve against the training iterations.

In the experiment, an initial learning rate of 0.001 and a dropout rate of 0.5 were set to keep the training process relatively stable. Fig. 7 illustrates the gradual improvement of the recognition accuracy and the decrease of the loss function against the training iterations. It can be seen that in the beginning stage, the training accuracy increased rapidly, indicating the fast convergence of the training process. Finally, after approximately 1500 iterations, the accuracy and loss function asymptotically approached to their limit, with trivial imperfectness due to imaging noise and numerical problems.

**Fig. 8.**
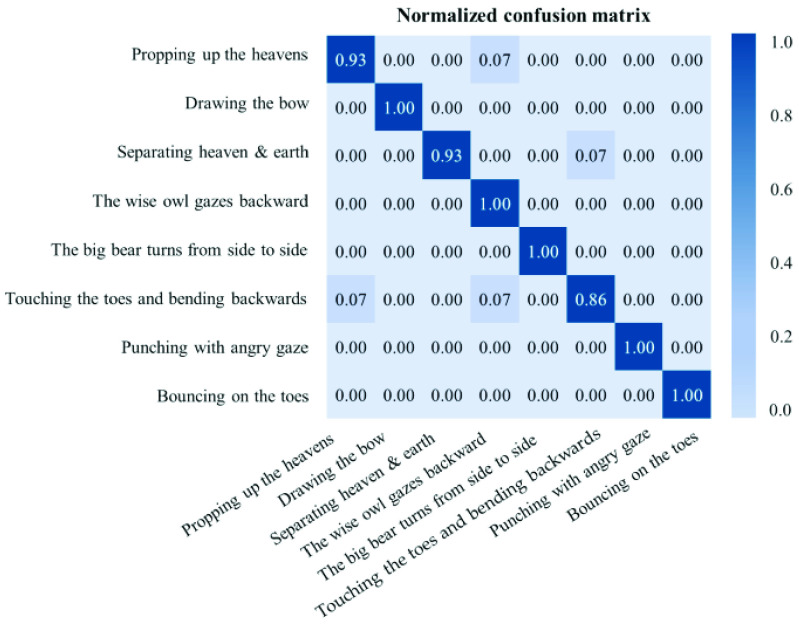
Normalized confusion matrix with predicted labels on the vertical axis and true labels on the horizontal axis.

After training for 200 epochs with 8 iterations per epoch, the Baduanjin action recognition model achieved 98.31% accuracy on the training data and 96.43% accuracy on the testing set. Fig. 8 shows the normalized confusion matrix for motion recognition on the testing set using this CNN-LSTM model. The high density along the diagonal indicates that most motions were correctly classified.

In Fig. 8, except for the pose of “Touching the toes and bending backward” in the test set, the classification performance of most poses reached or was very close to the perfection. In fact, for this specific pose, two cases were misclassified as “Propping up the heavens” and “The wise owl gazes backward” respectively, which thus reduced the accuracy of this pose to 85.71%. The reason for this abnor-mality can be attributed to the fact that these poses are actually very similar in the initial stage of the movement, in that all these three poses first require raising arms up in the same fashion while standing still.

On the other hand, for the method based on manually extracted motion features, as introduced in Sec II-B, nine typical joint angles and position trajectories were selected to distinguish different Baduanjin movements, according to the suggestions of professional physiotherapists. The experimental results show that these manually extracted features were only able to achieve 66.07% classification accuracy on the testing video dataset.

**Fig. 9.**
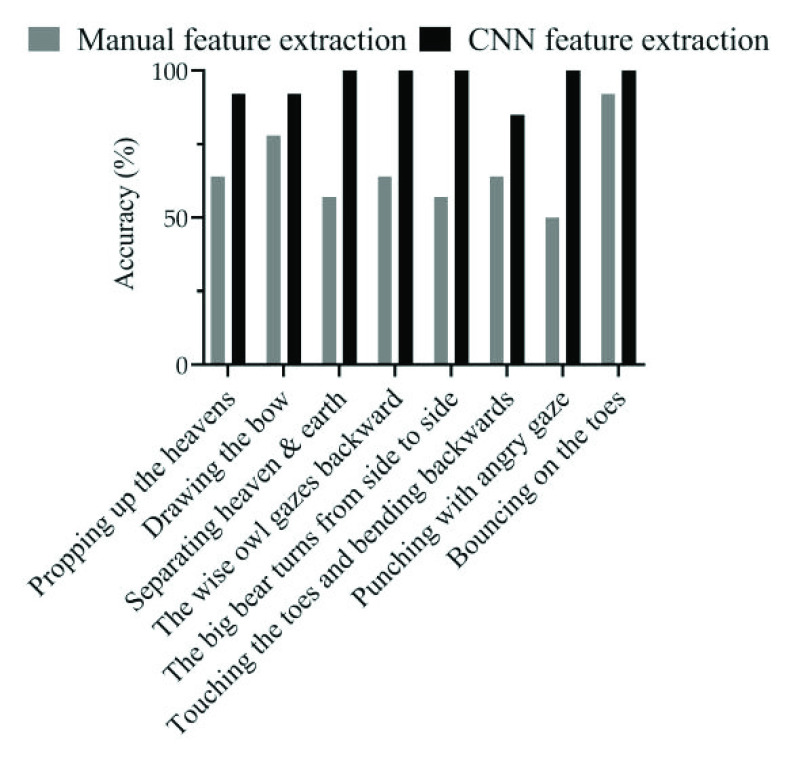
Comparison of the classification performances of the Baduanjin action recognition models respectively based on traditional manual feature extraction and CNN-LSTM.

The classification performances on the testing set of the two Baduanjin action recognition models respectively based on the traditional manual feature extraction and CNN-LSTM are finally compared in Fig. 9 and Table 3. It can be clearly seen that the overall accuracy of the method based on the traditional manual feature extraction is always lower than that of the CNN-LSTM based method. More specifically, according to Table 3, the accuracy of recognizing a single action was higher than 85.71% by the CNN-LSTM based method. Whereas, the lowest accuracy of recognizing a single action by the method based on traditional manual feature extraction was only 50%. This indicates that the features extracted by the VGG16 net are much more effective in uniquely representing the different poses than the artificial feature extraction method.

**TABLE 3:** Baduanjin Action Recognition Results From the Two Models with Different Feature Extraction Methods

No.	Pose name	Manual feature extraction	CNN feature extraction
1	Propping up the heavens	64.29 %	92.86 %
2	Drawing the bow	78.57 %	92.86 %
3	Separating heaven & earth	57.14 %	100.00 %
4	The wise owl gazes backward	64.29 %	100.00 %
5	The big bear turns from side to side	57.14 %	100.00 %
6	Touching the toes and bending backwards	64.29 %	85.71 %
7	Punching with angry gaze	50.00 %	100.00 %
8	Bouncing on the toes	92.86 %	100.00 %
	Total accuracy	66.07%	96.43%

## IV. Discussion

To deal with the complicated motions captured in a video format, we have proposed and tested in this work a combined CNN and LSTM model structure. The VGG16 convolution network is applied to extract the features of a pose snapshotted in every single picture. The time series of the pose features are then fed into the LSTM module, which models the dynamics of the poses in the video. The proposed CNN-LSTM method can thus model the complicated actions in the Baduanjin exercise.

By training and testing the method on the video data set (3:1 division at the video level) recorded from 18 Baduanjin practicers, the proposed model managed to recognize the Baduanjin actions with the average success rate of respectively 98.31% on the training data and 96.43% on the testing set. The results have therefore verified the effectiveness of this method.

The proposed CNN-LSTM method has been compared with the traditional method based on geometric feature extraction. For fair comparison, the time series of the multidimensional geometric features extracted from every picture in the video, including nine typical joint angles and position trajectories, are also processed by a LSTM module. However, it turned out in the testing that the method based on these manually extracted features was only able to achieve an average success rate of 66.07% on the testing video dataset, and always performed worse than the CNN-LSTM based method in recognizing all the eight different actions in Baduanjin. Through this comparison, the effectiveness of the VGG16 net in extracting the abstract motion features to uniquely represent the different poses in the video has been clearly verified.

The proposed method still contains a few limitations. First, the convergence rate is not high. In the training experiment, with the initial learning rate of 0.001 and the dropout rate of 0.5, the accuracy and loss function converged after approximately 1500 iterations. Second, the method does not perform well for the different actions containing similar poses, indicating the sensitivity of the method needs to be further improved. The typical example is the action of “Touching the toes and bending backward”, which can be misclassified as “Propping up the heavens” or “The wise owl gazes backward”, because all these three actions first require rising arms up while standing still in the same fashion.

On the other hand, a major advantage of convolutional neural networks over traditional methods is that they can automatically learn multi-scale information. However, the capability of representing multi-scale information by a VGG network is relatively limited compared with other recently improved CNN models, which may lead to the aforementioned limitation in the sensitivity of the proposed method. The examples of the improved CNN models include Res2Net and DenseNet, which can further enhance multi-scale representation through skip layer linking, and can thus outperform VGG networks in multi-scale feature expression [44].

## V. Conclusion

In this work, a combined VGG16 type of CNN and LSTM model has been developed and investigated for identifying human actions from video data, which has a wide applicability in many newly emerging fields to improve human wellbeing, such as intelligent rehabilitation, health surveillance and sports skill assessment. More specifically, the method has been applied on recognizing the sequence of complicated actions performed by practicers of a traditional Chinese exercise (ie, Baduanjin). Its performance has been verified by the high classification accuracy on the testing video dataset. Moreover, this method has been compared with the LSTM model, which takes as inputs the positional and angular motion features extracted from the detected skeleton data via Openpose. The results have clearly demonstrated that the image features extracted by the CNN module are more effective than the geometric features on improving the final classification accuracy of the LSTM model. Therefore, the proposed CNN-LSTM based method can be a useful tool in recognizing the actions of Baduanjin and other similar exercises, and can whereby help the practicers improve their skills.

A potential extension of the current work is to implement and test the proposed CNN-LSTM based method in other exercises with even faster movements, e.g., boxing and badminton. For these exercises, faster imaging solutions should be developed. Another direction of the future work is to further improve the sensitivity of the proposed model by incorporating the multi-scale representation functionalities of Res2Net, or to implement more advanced models, such as TS-LSTM.
